# *Ginkgo biloba* leaf extract (EGb-761) elicits neuroprotection against cerebral ischemia/reperfusion injury by enhancement of autophagy flux in neurons in the penumbra

**DOI:** 10.22038/IJBMS.2021.46318.10694

**Published:** 2021-08

**Authors:** Deng Yihao, Guo Tao, Wu Zhiyuan, Zhao Xiaoming, Dong Lingling, He Hongyun

**Affiliations:** 1 Department of Basic Medicine, Medical School, Kunming University of Science and Technology, Kunming 650500, China

**Keywords:** Autophagy, Enhancement, Ginkgo biloba, Ischemic stroke, Neuroprotection

## Abstract

**Objective(s):**

*Ginkgo biloba *leaf extract (EGb-761) injection has been widely used as adjuvant therapy for cerebral stroke in China. However, its underlying pharmacological mechanism is not completely understood. The present study aimed to investigate whether the therapeutic effects of EGb-761 are exerted by modulating autophagy flux.

**Materials and Methods:**

Ischemic cerebral stroke was prepared in male Sprague-Dawley rats by middle cerebral artery occlusion (MCAO) followed by reperfusion. The MCAO/reperfusion rats were then treated with EGb-761 injection once daily for 7 days. Thereafter, the brain tissues in the ischemic penumbra were obtained to detect the key proteins in the autophagic/lysosomal pathway with Beclin1, LC3, (SQSTM1)/p62, ubiquitin, LAMP-1, cathepsin B, and cathepsin D antibodies by western blot and immunofluorescence. Meanwhile, the infarct volume, neurological deficits, and neuronal apoptosis were assessed to evaluate the therapeutic outcomes.

**Results:**

The results illustrated that EGb-761 treatment was not only able to promote the autophagic activities of Beclin1 and LC3-II in neurons, but also could enhance the autophagic clearance, as indicated by reinforced lysosomal activities of LAMP-1, cathepsin B, and cathepsin D, as well as alleviating autophagic accumulation of ubiquitin and insoluble p62 in the MCAO+EGb-761 group, compared with those in the MCAO+saline group. Meanwhile, cerebral ischemia-induced neurological deficits, infarct volume, and neuronal apoptosis were significantly attenuated by 7 days of EGb-761 therapy.

**Conclusion:**

Our data suggest that EGb-761 injection can elicit a neuroprotective efficacy against MCAO/reperfusion injury, and this neuroprotection may be exerted by enhancement of autophagy flux in neurons in the ischemic penumbra.

## Introduction

Ischemic stroke is a leading cause of permanent disability and the second most common cause of death throughout the world (1). There are approximately 15 million people who suffer a stroke each year, and most stroke survivors are suffering from serious neurological impairments (2). Moreover, a huge number of stroke patients presents great economic and social burdens (3). After cerebral ischemia, diverse pathological mechanisms are triggered, including inflammatory response, apoptosis, calcium overload, oxidative stress, and excitotoxic injury which collectively result in cellular death within the brain (4-6). The only U.S. Food and Drug Administration (FDA)-approved drug for the treatment of ischemic stroke is the tissue-type plasminogen activator (tPA), but the narrow therapeutic time window within 4.5 hr greatly limits its application [7), leading to only about 5% of the patients benefiting from this treatment. Neuroprotective components have been previously identified for stroke treatment, such as antiplatelet agents, fibrinolytic agents, and anticoagulants (8), but their beneficial effects are still very limited. Thus, novel drugs are urgently needed for patients suffering from ischemic stroke.

*Ginkgo* *biloba* is a traditional Chinese herb and widely used to treat brain disorders for thousands of years (9). Herbal medicines generally constitute tens of or hundreds of components. Therefore, identifying and isolating the effective components from the herbal medicines is essential to their application (10). For this reason, a standardized formulation of *Gingko biloba* leaf extract (EGb-761^®^) has been determined to contain ~24% flavone glycosides, ~6% terpene lactones consisting of 2.6%-3.2% bilobalide, and 2.8%-3.4% ginkgolides (A, B, and C), and less than 5 ppm ginkgolic acid (11). The flavonoids and terpene trilactones are generally considered to be the bioactive components in EGb-761 (12). EGb-761 has been used to treat various ailments, such as Alzheimer’s disease, dementia, cerebrovascular insufficiency, and cerebral infarction (13, 14), etc. Insufficiency of blood flow, ischemia, and neurodegeneration are three primary pathological elements that deteriorate the neurological injury following ischemic stroke. Animal studies have shown that EGb-761 was not only able to increase cerebral blood flow after stroke (15), but also could alleviate the reperfusion injury by enhancing free-radical scavenging after restoration of blood flow (16). Meanwhile, EGb-761 has been demonstrated to attenuate excitotoxic injury by inhibition of ischemia-induced glutamate release (17). Besides, EGb-761 was shown to reduce infarct volume, attenuate apoptosis, and improve neurological function (18). Moreover, clinical trials have also reported the neuroprotective effect of EGb-761 in the treatment of ischemic stroke (19). However, these animal studies and clinical researches only displayed its therapeutic outcomes, but seldom elucidated how EGb-761 elicited the neuroprotection after stroke.

Autophagy is initially known as a cellular protective mechanism by which damaged organelles, superfluous cytoplasmic components, nonfunctional proteins, and certain pathogens are delivered to lysosomes for degradation (20). Autophagy plays a cytoprotective role in cell survival by retaining ATP source, and by reclaiming cytoplasmic constituents and essential nutrients under physiological conditions (21). It can be prominently activated by ischemia following stroke (22), but the role of autophagy in cerebral stroke is continuously controversial (23). Increasing evidence demonstrated the neuroprotective effects of autophagy in ischemic stroke (24). However, numerous studies also showed the detrimental role of autophagy in cerebral ischemia (25). In fact, autophagy is metabolic machinery containing several consecutive processes of autophagosome formation, fusion of the autophagosome with lysosomes, and autophagic degradation in autophagolysosomes (26). The integral state of these autophagic processes can be termed “autophagy flux”, being very important for restoration of cellular homeostasis (27). To achieve the function of metabolic clearance, the contents within autophagosomes need to be efficiently digested by lysosomes (28). Thus, autophagic degradation in lysosomes is a critical step for the final clearance of autophagic cargoes, and the lysosomal function, therefore, plays a key role in autophagy. A recent study illustrated that lysosomal dysfunction-caused autophagy flux impairment might be the main pathogenesis of neurological injury after stroke (29). Therefore, we especially focused on the state of lysosomal capacity besides autophagic activity to monitor the integral autophagy flux after ischemic stroke in our study. 

EGb-761 has been shown to elicit neuroprotective effects against ischemic injury after stroke. Meanwhile, autophagy has been validated to extensively participate in the pathological processes of cerebral stroke. We discussed whether the neuroprotection elicited by EGb-761 treatment was induced by modulation of autophagic/lysosomal pathway after ischemic stroke. Therefore, this study was first to uncover whether EGb-761 could alleviate neurological injury after ischemic stroke, and then investigated the efficacy of EGb-761 on autophagy flux. The results demonstrated that 7 days of EGb-761 treatment significantly alleviated the neurological injury after ischemic stroke. Moreover, EGb-761 therapy was not only able to promote the autophagic activities in neurons at the ischemic penumbra, but also could restore the ischemic stroke-caused lysosomal dysfunction and consequently attenuated the autophagic accumulation. Based on these data, we concluded that EGb-761 elicited neuroprotection against cerebral ischemia/reperfusion injury by enhancement of autophagy flux. 

## Materials and Methods

***Experimental animals***

Pathogen-free male Sprague-Dawley rats were purchased from Hunan Slac Laboratory Animal Corporation (certificated number: SCXK2016-0002). The rats were 9–10 weeks old weighing 250 g–280 g and fed with animal welfare practices. The animals were given free access to fresh water and food under controlled conditions with 21±1 °C and 60±5% relative humidity. Surgeries were performed under anesthesia with 10% chloral hydrate to minimize the suffering. All animal experiments were approved by the animal experiment committee of Kunming University of Science and Technology (Kunming, China). A total of 66 rats were recruited, 12 rats died during or after the operation (mortality was approximately 18.18%). 54 animals were included in this study: 18 rats were used to evaluate neurological deficit and infarct volume, 18 animals were for TdT-mediated dUTP Nick-End Labeling (TUNEL) and immunofluorescence, and the remaining 18 rats were used to detect the proteins in autophagic/lysosomal pathway by western blot. 

***Preparation of middle cerebral artery occlusion (MCAO)/reperfusion in rats***

A rat model of transient ischemic stroke was prepared under anesthesia with 10% chloral hydrate (400 mg/Kg, IP). The left external carotid artery (ECA), internal carotid artery (ICA), and common carotid artery (CCA) were isolated from adjacent nerves and muscles, respectively. A 4-0 nylon monofilament (Beijing Xinong Biotechnology Co., Ltd, Beijing, China) was first inserted into ECA through a mini-incision on ECA, and then returned into ICA from CCA, and finally advanced 20±1 mm into the middle cerebral artery. The nylon monofilament was coated with a round polylysine tip that was approximately 0.36 mm, being similar to the diameter of the middle cerebral artery of SD rats weighing 250–280g. Therefore, the insertion with a nylon monofilament could lead to complete occlusion of the middle cerebral artery (MCAO). After 90 min of MCAO, the nylon monofilament was gently withdrawn for reperfusion. The method to prepare the MCAO/reperfusion rat model was also based on our previous study (30).

***EGb-761 treatment and experimental groups***

The MCAO/reperfusion rats were randomly divided into 3 groups: MCAO+EGb group, the rats were treated with EGb-761 injection (Zhonghao International Co. Ltd., China, 10 mg/Kg) by intraperitoneal injection (IP) once daily for 7 days after onset of reperfusion. Because a study (31) has shown that a dose of 10 mg/Kg of EGb-761 treatment could significantly alleviate cerebral ischemia-caused neurological injury, so we determined this administration dose to investigate its neuroprotective mechanism in this study; MCAO+saline group, the animals were given the same volume of physiological saline instead of EGb-761 injection; Sham group, the sham operation rats were administrated the same volume of physiological saline.

***Protein isolation and western blot***

The MCAO/reperfusion rats were sacrificed under anesthesia by 10% chloral hydrate (400 mg/Kg, IP) after 7 days of EGb-761 treatment. The brain tissues in the penumbra were rapidly isolated on ice, and homogenized by abrasiveness, and were then added into a RIPA buffer (containing 50 mM Tris pH 7.4, 150 mM NaCl, 1% TritonX-100, 1% sodium deoxycholate, and 0.1% SDS, Beijing BLKW Biotechnology Co., Ltd, Beijing, China) for 45 min at room temperature. After 12,000g centrifugation for 15 min at 4 °C, the supernatants of the penumbral tissues were obtained. Meanwhile, the insoluble proteins in the lysates were obtained with a kit of Inclusion Body Solubilization Buffer (Sangon Biotechnology, Shanghai, China). SDS-PAGE gel electrophoresis was used to separate the proteins in the supernatants. The proteins were then transferred into polyvinylidene fluoride (PVDF) membranes (Millipore, Billerica, MA, USA). Thereafter, the PVDF membranes were blocked with 10% nonfat milk at room temperature for 2 hr and then washed with PBST (PBS containing 0.1% polysorbate 20). Rabbit antibodies against rat LC3 (1:1000 in PBST, Cell Signaling Technology, Danvers, MA, USA), Beclin1 (1:1000, Cell Signaling Technology), SQSTM1/p62 (Cell Signaling Technology, 1:1000), Cathepsin B (Santa Cruz Biotechnology, Dallas, TX, USA, 1:1000), Cathepsin D (Santa Cruz Biotechnology, 1:1000), LAMP-1 (ABclonal Technology, Wuhan, China, 1:700), ubiquitin (ABclonal Technology, 1:800) and beta-actin (1:10000, β-actin, Abcam, Cambridge, UK) were incubated at 4 °C overnight. After washing, the horseradish peroxidase (HRP)-conjugated anti-rabbit IgG (1:5000, Invitrogen, Shanghai, China) was labeled at room temperature for 1 hr. After 2 hr of washing under shaking, the reaction was amplified by electrochemiluminescence (ECL). The optical density of the bands was quantified by Image J software. The fluorescence density was normalized to β-actin.

***Immunofluorescence***

The rats were sacrificed under anesthesia after 7 days of EGb-761 treatment. The brains were quickly removed and dehydrated in a 30% sucrose solution (Solarbio Science & Technology Co., Ltd, Beijing, China) for 24 hr, and were coronally sliced into sections of 20 μm of thickness with a freezing microtome (SLEE, Mainz, Germany). The brain sections were washed with 0.01M PBS and were permeabilized with Triton X-100 (0.2% in PBS) for 15 min. To block the non-specific reaction, the sections were dealt with BSA (10% in PBS, Sigma, St. Louis, MS, USA) for 1 hr. After that, rabbit antibodies against rat LC3 (1:400, Cell Signaling Technology, Danvers, MA, USA), Iba-1(1:400, Abcam), GFAP (1:400, Cell Signaling Technology), p62 (1:400, Cell Signaling Technology), mouse antibody against rat NeuN (1:400, Abcam, Cambridge, UK), LAMP-1 (1:200, ABclonal Technology, Wuhan, China) and Cathepsin B (1:400, Santa Cruz Biotechnology, Dallas, TX, USA) were added for incubation at room temperature for 4 hr. After a washing step, the Alexa Fluor-594-conjugated anti-rabbit IgG (1:800, Invitrogen, Shanghai, China) and Alexa Fluor-488-conjugated anti-mouse IgG (1:800, Invitrogen) were labeled for 2 hr in the dark. After washing, 4’, 6-diamidino-2-phenylindole (DAPI, 1:1000, Cell Signaling Technology) were added to counterstain in the dark for 5 min. After a washing step, the reaction was detected with a fluorescent microscope (Nikon Instruments Co., Ltd., Tokyo, Japan). Results were expressed as percentages of positive cells. Under high magnification (×400), the number of reaction-positive cells and the total number of cells were counted in 10 randomly selected penumbral areas in each section, respectively; five sections must be counted from each sample. 

***Detection of neuronal apoptosis***

The 20 μm-thickness brain sections were prepared as mentioned above. The sections were first stained by immunofluorescence with a primary mouse antibody against rat NeuN (a neuron marker, 1:400, Abcam, Cambridge, UK) and second antibody of Alexa Fluor-488-conjugated anti-mouse IgG (1:800, Invitrogen, Shanghai, China). After immunofluorescence, the brain sections were then stained with TdT-mediated dUTP Nick-End Labeling (TUNEL) to detect neuronal apoptosis at the penumbra. The TUNEL staining was performed according to instructions provided by the TUNEL kit (Beyotime Biotechnology Co., Ltd, Shanghai, China). Finally, the sections were counterstained with DAPI (1:1000, Cell Signaling Technology). The results were expressed as percentages of TUNEL-positive neurons. Under high magnification (×400), the number of TUNEL-positive neurons and the total number of neurons were counted in 10 randomly selected penumbral areas in each section, respectively; five sections must be counted from each sample. 

***Modified neurological severity score (mNSS)***

Neurological deficit was scored by a modified neurological severity score (mNSS) test after 7 days of EGb-761 treatment, according to a previous study (32). The mNSS test included 4 parts: Motor function including abnormal movement and muscle status; Sensory function including proprioceptive, tactile, and visual deficits; Reflex evaluation; and test of balance ability. The total score was 18, and no neurological deficit scored 0. Therefore, a higher score indicated a more severe neurological deficit.

***Detection of brain infarct volume***

The rats were sacrificed under anesthesia with 10% chloral hydrate (400 mg/Kg, IP). The brains were quickly removed on ice and immediately frozen at -20 °C for 20 min and were then coronally sliced into 2 mm-thickness sections. The brain sections were immediately stained with triphenyl tetrazolium chloride (TTC, 2% in PBS, Solarbio Science & Technology Co., Ltd, Beijing, China) for 30 min at 37 °C. By TTC staining, the normal brain tissues were shown red and the infarct tissues were pale. An imaging software (Adobe Photoshop CS6) was used to calculate the infarct volume. The result was determined by infarction rate (%) = A°/A’ × 100%, A’ was the volume of the ipsilateral hemisphere, A° represented the infarct volume.

***Statistical analysis***

All data were presented as means ± standard error of the mean (SEM). Statistical differences were evaluated by one-way analysis of variance (ANOVA) followed by Dunnett’s test. *P*<0.05 was considered statistically significant.

## Results

EGb-761 significantly promoted autophagic activity and attenuated the accumulation of autophagic cargoes 

**Figure 1:**
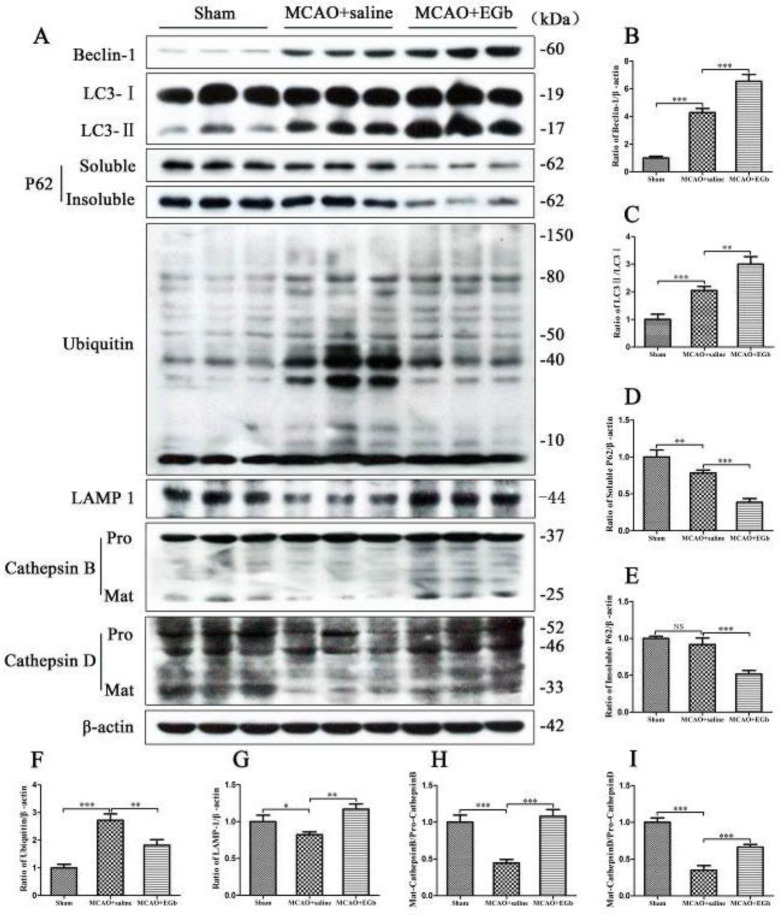
Proteins in the autophagic/lysosomal pathway were detected by western blot after 7 days of EGb-761 treatment following MCAO/reperfusion. The autophagic levels of Beclin-1 (Figures A, B) and LC3-II (Figures A, C), and the lysosomal activities of LAMP-1 (Figures A, G), cathepsin B (Figures A, H), and cathepsin D (Figures A, I) in the penumbra were significantly elevated by 7 days of EGb-761 treatment. Correspondingly, the autophagic substrates were dramatically reduced by EGb-761 therapy in the MCAO+EGb-761 group, compared with those in the MCAO+saline group. n=6, NS: not significant, * *P*<0.05, **P<0.01, ***P<0.001. Pro: precursor form, Mat: mature form

The rats were immediately treated with EGb-761 for 7 days following MCAO/reperfusion, the brain tissues in the penumbra were obtained to detect the proteins in the autophagic/lysosomal pathway by western blot. The results showed that both Beclin1 expression (Figures 1A, B) and the ratio of LC3-II/LC3-I (Figures 1A, C) were significantly elevated by EGb-761 treatment in the MCAO+EGb-761 group, compared with those in the MCAO+saline group. Moreover, EGb-761 therapy significantly reinforced the lysosomal activities of LAMP-1 (Figures 1A, G), cathepsin B (Figures 1A, H), and cathepsin D (Figures 1A, I), and prominently reduced the autophagic substrates of ubiquitin (Figures 1A, F) and insoluble p62 (Figures 1A, E). 

***EGb-761 treatment-promoted autophagic activity mainly occurred in neurons***

**Figure 2:**
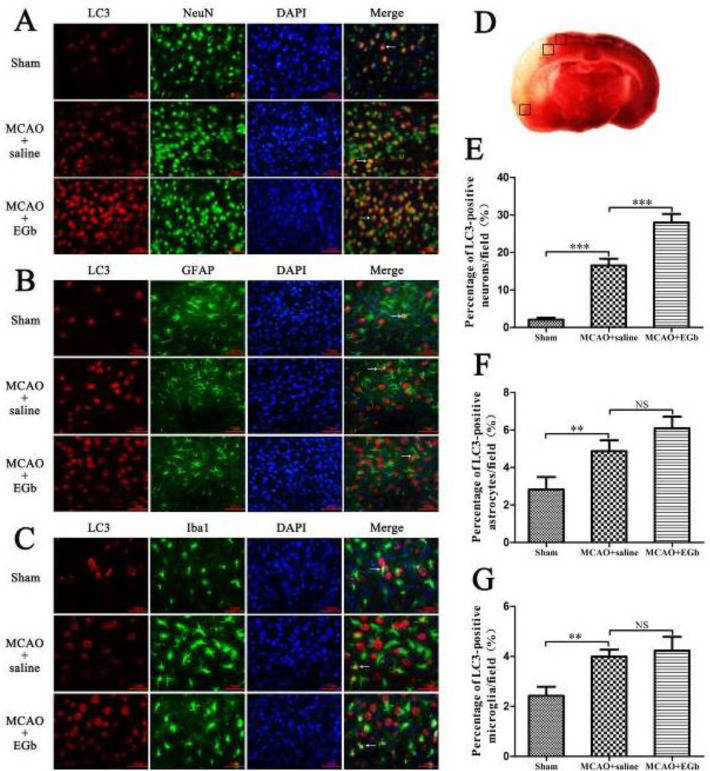
The EGb-761 treatment-changed autophagic activity was further verified by double immunofluorescence and for cellular localization. The percentages of LC3-NeuN (Figure A), LC3-GFAP (Figure B), and LC3-Iba1-positive cells (Figure C) in the ischemic penumbra were measured by double immunofluorescence. The result demonstrated that the ratio of LC3-NeuN-positive cells (Figure E) was obviously improved by EGb-761 treatment, compared with that in the MCAO+saline group. Comparatively, the percentages of both LC3-GFAP (Figure F) and LC3-Iba1-positive cells (Figure G) in the MCAO+EGb-761 group were similar to those in the MCAO+saline group. LC3: a marker of autophagosome formation; NeuN: a neuron indicator; Iba-1: a microglia marker; GFAP: an astrocyte indicator. Bar=50 µm. The arrows indicated the LC3-positive cells. The black squares in Figure D showed the selected penumbral area (cortical brain tissue) for counting LC3-positive cells. n=6, NS: not significant, **P<0.01, ***P<0.001

Double immunofluorescence was performed to investigate the cellular localization of EGb-761 treatment-altered autophagic activity with antibodies of LC3-II (an autophagy indicator), NeuN (a neuron marker), Iba-1 (a microglia indicator), and GFAP (an astrocyte marker). The results illustrated that the percentage of LC3-positive neurons (Figures 2A, E) was dramatically promoted by EGb-761 treatment in the MCAO+EGb-761 group, compared with that in the MCAO+saline group. However, the ratios of LC3-positive astrocytes (Figures 2B, F) and microglia (Figures 2C, G) in MCAO+EGb-761 were similar to those in the MCAO+saline group. 

***EGb-761 therapy attenuated the ischemic stroke-induced lysosomal dysfunction***

**Figure 3:**
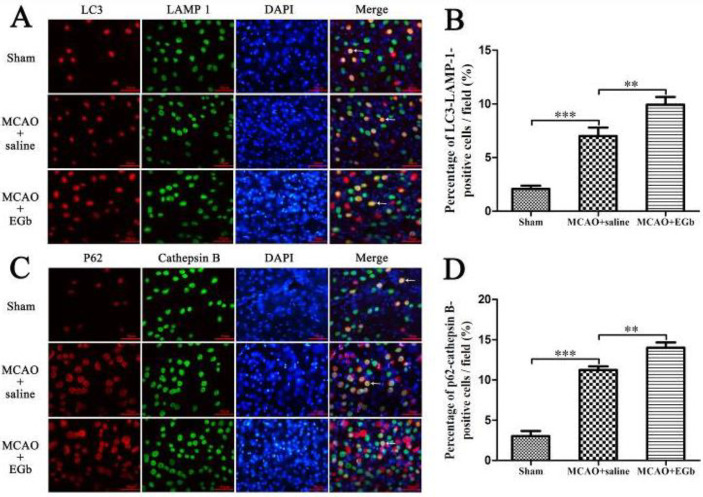
Double immunofluorescence was performed to evaluate lysosomal function. After 7 days of EGb-761 treatment after MCAO/reperfusion, the ratios of LC3-II-LAMP-1 (Figures A, B) and p62-cathepsin B-positive cells (Figures C, D) in the penumbra were markedly promoted, compared with those in the MCAO group. This implied that the ischemic stroke-caused lysosomal dysfunction could be restored by EGb-761 therapy. LC3: a marker of the mature autophagosome, LAMP-1: an indicator of lysosomal activation, p62: a recruiting protein of autophagic substrates, cathepsin B: a main protease of lysosomes. Bar=50 µm. n=6, **P<0.01, ***P<0.001

Double immunofluorescence was performed to evaluate lysosomal function with antibodies of LC3-II (an indicator of autophagosome), LAMP-1 (a marker of lysosomal activation), p62 (a recruiting protein of autophagic substrates), and cathepsin B (a main protease of lysosomes). The results demonstrated that the percentages of LC3-II-LAMP-1 (Figures 3A, B) and p62-cathepsin B-positive cells (Figures 3C, D) in the ischemic penumbra were significantly elevated by EGb-761 treatment, compared with those in the MCAO+saline group. This indicated that the ischemic stroke-caused lysosomal dysfunction could be restored by EGb-761 therapy.

***EGb-761 dramatically alleviated the cerebral ischemia-caused neurological deficit***

**Figure 4.**
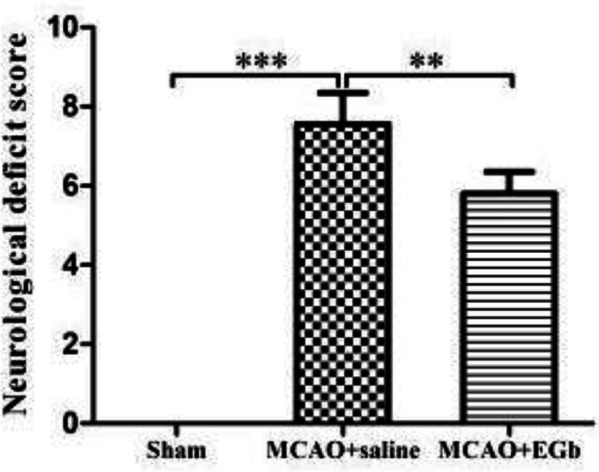
mNNS test was assessed to evaluate the therapeutic effect of EGb-761 treatment after MCAO/reperfusion. After 7 days of EGb-761 therapy, the ischemic stroke-induced neurological deficit was significantly alleviated in the MCAO+EGb-761 group, compared with that in the MCAO+saline group. No neurological deficiency was observed in the sham group. n=6, **P<0.01, ***P<0.001

The mNSS test was assessed to investigate the effect of EGb-761 therapy on neurological deficit after MCAO/reperfusion. A serious neurological deficit was observed in the MCAO/reperfusion rats, compared with those in sham surgery animals. After 7 days of EGb-761 therapy, the neurological deficit was obviously alleviated in the MCAO+EGb-761 group, compared with that in the MCAO+saline group (Figure 4).

***Ischemic stroke-caused infarction could be prominently attenuated by EGb-761***

**Figure 5:**
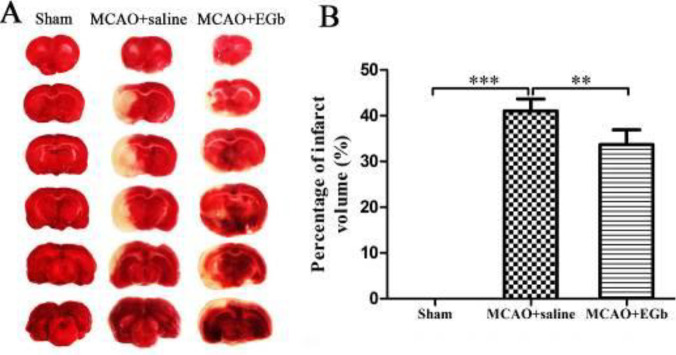
Infarct volume was measured by TTC staining after 7 days of EGb-761 treatment following MCAO/reperfusion. The infarct volume was significantly attenuated by EGb-761 therapy in the MCAO+EGb-761 group (Figure A), compared with that in the MCAO+saline group (Figure B). No infarction was detected in the sham group. n=6, **P<0.01, ***P<0.001

The infarct volume was measured by TTC staining after 7 days of EGb-761 treatment following MCAO/reperfusion (Figure 5A). The results (Figure 5B) illustrated that the infarct volume could be significantly attenuated by 7 days of EGb-761 treatment in the MCAO+EGb-761 group, compared with that in the MCAO+saline group. 

***Neuronal apoptosis in the penumbra was markedly reduced by EGb-761 treatment***

**Figure 6:**
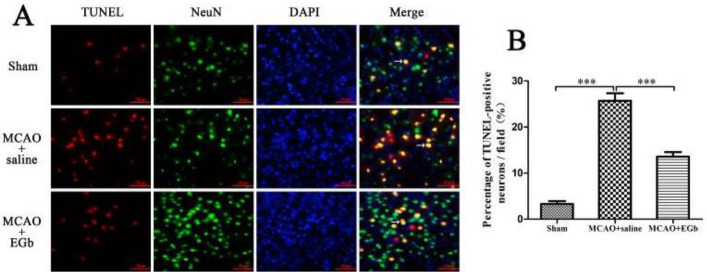
Neuronal apoptosis in the penumbra was detected by TUNEL staining compounded with immunofluorescence. After 7 days of EGb-761 treatment following MCAO/reperfusion, the neuronal apoptosis in the ischemic penumbra was detected by immunofluorescence compounded with TUNEL staining (Figure A). The result (Figure B) indicated that the percentage of TUNEL-NeuN-positive cells in the MCAO+EGb-761 group was significantly lower than that in the MCAO+saline group. The TUNEL-positive cells (red stained) showed apoptotic cells, the NeunN-positive cells (green-labeled) indicated neurons. The arrows indicated the apoptotic neurons. Bar=50 µm. n=6, ***P<0.001

The neuronal apoptosis at the penumbra was detected by TUNEL staining compounded with immunofluorescence after EGb-761 treatment following MCAO/reperfusion (Figure 6A). The result (Figure 6B) showed that the percentage of TUNEL-positive neurons was significantly higher than that in the sham group. After 7 days of EGb-761 treatment, the ratio of apoptotic neurons was markedly reduced in the MCAO+EGb-761 group, compared with that in the MCAO+saline group. 

## Discussion

*Ginkgo* *biloba* extract (EGb) is a compound drug derived from *Ginkgo biloba* trees native to China. It has a long history of use in traditional Chinese medicine (TCM) to treat multiple diseases, such as cardiocerebrovascular insufficiency (33). Both animal studies and clinical trials confirmed that EGb was able to elicit noticeable neuroprotection against ischemic brain injury (34, 35). Based on its biological properties, an injection preparation of standardized *Ginkgo biloba* extract (EGb-761) has been extensively used as an adjunctive treatment for clinical stroke in China (36). Previous studies revealed that the efficacy of EGb-761 to alleviate post-stroke damage was exerted by reduction of infarct volume, inhibition of apoptosis, improvement of neurological function, enhancement of anti-oxidant enzymes, and mitigation of excitotoxic injury, etc (37, 38). However, the detailed therapeutic mechanisms of EGb-761 for stroke treatment were not completely understood. 

Numerous studies have uncovered that autophagy was prominently activated by the ischemia following stroke. Moreover, the autophagic level had a striking impact on the pathological development of ischemic stroke (39). The roles that autophagy played in ischemic stroke were controversial among studies, but the degree of ischemia, research designs, detection methods, and experimental models might be the important elements to determine whether autophagy was beneficial or harmful after stroke (40, 41). Studies showed that acute stroke or severe ischemia might lead to “excessive autophagy”, and thereby aggravate neurological damage (42). Relative, chronic or mild ischemia likely induced an “appropriate autophagy”, thereby protecting cells by scavenging damaged tissues and debris (43). Unfortunately, how much autophagy was excessive or moderate was confused, leading to contradictory and even opposite conclusions being drawn concerning the role of autophagy in stroke among investigations (22, 23). However, the main fault of these previous studies was that they evaluated the role of autophagy only by investigation of autophagic activity. Because integral autophagy contained several consecutive processes of autophagosome formation and maturation, autophagolysosome formation by fusion of the autophagosome with lysosomes, and autophagic degradation and clearance in autophagolysosomes (44), so it was not convincing to determine the role of autophagy in stroke only by evaluation of the autophagic activity. For this reason, the autophagy initiation and autophagosome formation were respectively detected with Beclin1 and LC3 antibodies (45), the lysosomal activity was evaluated by LAMP-1 (46), cathepsin B, and cathepsin D (47), and the content of autophagic cargoes was assessed by p62 (48) and ubiquitin (49) in the present study. Thus, the integral autophagic processes could be completely monitored to investigate the efficacy of EGb-761 therapy on the autophagic/lysosomal pathway (autophagy flux).

Our study demonstrated that ischemic stroke led to serious neurological deficit (Figure 3), cerebral infarction (Figure 4), and neuronal apoptosis (Figure 5) in the MCAO+saline group. The western blot (Figure 1A) demonstrated that the declined expression of LAMP-1 was accompanied by attenuated activities of cathepsin B and cathepsin D in the MCAO+saline group, compared with those in the sham group. This indicated that the lysosomal function was repressed by the ischemia after stroke. Moreover, the ischemic stroke-weakened lysosomal capacity resulted in accumulation of autophagic substrates, as illustrated by the improved expressions of Beclin-1 (Figure 1B), LC3-II (Figure 1C), insoluble p62 (Figure 1E), and ubiquitin (Figure 1F). This outcome was also consistent with the reported studies which suggested that lysosomal dysfunction was important pathogenesis of ischemic stroke (29, 50). After 7 days of EGb-761 treatment, the ischemic stroke-caused neurological injury was markedly alleviated. This result was also similar to the reported studies (51, 52). To investigate whether this neuroprotection was elicited by modulating the autophagic/lysosomal pathway, we further explored the effect of EGb-761 on autophagy flux. The result showed that the lysosomal dysfunction could be dramatically mitigated, as reflected by promoted expressions of LAMP-1 (Figure 1G), cathepsin B (Figure 1H), and cathepsin D (Figure 1I) after EGb-761 therapy. The expressions of Beclin-1 and LC3-II at the penumbra were elevated by EGb-761 treatment, but the levels of insoluble p62 and ubiquitin were contrarily reduced in the MCAO+EGb-761 group, compared with those in the MCAO+saline group. This suggested that EGb-761 treatment improved autophagic activity which inevitably increased the production of autophagic substrates, but the increased autophagic cargoes could be effectively degraded by EGb-761-enhanced lysosomal capacity. Furthermore, the immunofluorescence (Figures 2A-C) demonstrated that EGb-761-promoted autophagic activity was mainly displayed in neurons at the ischemic penumbra (Figures 2E-G). Besides, the double immunofluorescence illustrated (Figures 3A, C) that the percentages of LC3-II-LAMP-1 (Figure 3B) and p62-cathepsin B-positive cells (Figure 3D) were significantly promoted by EGb-761 treatment, compared with those in the MCAO+saline group. These outcomes further confirmed that the ischemic stroke-caused lysosomal dysfunction could be restored by EGb-761 therapy. 

## Conclusion

Our data showed that the infarct volume, neurological deficit, and neuronal apoptosis were significantly attenuated by 7 days of EGb-761 treatment after ischemic stroke. Meanwhile, our results indicated that EGb-761 therapy was not only able to promote autophagic activity in neurons at the ischemic penumbra, but also could enhance the autophagic clearance by reinforcing the lysosomal function after MCAO/reperfusion. Therefore, we concluded that the EGb-761-elicited neuroprotection was exerted by facilitation of autophagic/lysosomal pathway (autophagy flux) after ischemic stroke. 
